# Enhancing *Eyringpy*: Accurate Rate Constants with Canonical Variational Transition State Theory and the Hindered Rotor Model

**DOI:** 10.1021/acs.jctc.4c00926

**Published:** 2024-11-07

**Authors:** Eugenia Dzib, Alan Quintal, Gabriel Merino

**Affiliations:** † Departamento de Física Aplicada, Centro de Investigación y de Estudios Avanzados, Km. 6 Antigua carretera a Progreso Apdo. Postal 73, Cordemex, 97310 Mérida, Mexico

## Abstract

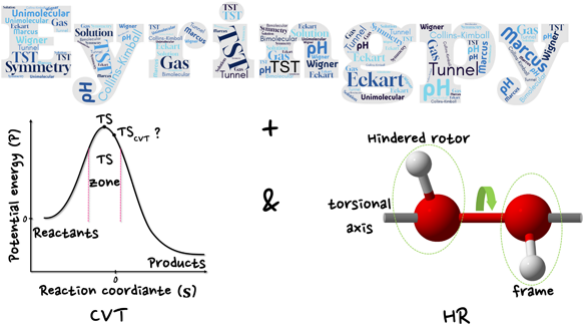

The recrossing effect and hindered rotations can lead to significant inaccuracies in rate constant calculations using transition state theory and the harmonic oscillator approximation. To address these issues, we enhanced *Eyringpy*, a *Python*-based computational tool, by integrating the canonical variational transition state theory (CVT) and the hindered rotor model, effectively mitigating the limitations of traditional methods. CVT rate constants are calculated using electronic structure data from nonstationary points along the minimum-energy path. Additionally, we developed an algorithm based on reaction force analysis to autonomously select relevant nonstationary points. Torsions are modeled using one-dimensional hindered rotor approaches proposed by Pitzer-Gwinn and Ayala-Schlegel.

## Introduction

Nearly 90 years after its formulation, transition state theory (TST)^1,2^ has become a widely applied tool in chemical kinetics, mainly due to advances in electronic structure computations. TST’s popularity arises from its ability to calculate rate constants directly from the thermodynamic properties of reactants and the transition state (TS),^3^ making it useful for a broad range of reactions. Although simple in principle, TST yields accurate results when activation energies and structural properties are derived from precise electronic structure methods.^4−8^ For instance, for many gas-phase reactions, TST rate constants are accurate within a factor of 2.^7,8^ However, TST has limitations.

A key limitation of TST is its assumption that reactant-side trajectories transverse the potential energy surface’s (PES) dividing surface only once (nonrecrossing assumption).^3,9−12^ This contradicts the inherent nature of chemical systems, where recrossing is common.^9,13,14^ Consequently, TST often overestimates rate constants, especially for reactions with low activation energies or at high temperatures.^13,15,16^ To minimize recrossing, the dividing surface must be placed at the “dynamical bottleneck”, a principle that is the foundation of the canonical variational transition state theory (also called canonical variational theory, CVT).^9−11,17^

Another source of uncertainty in TST arises from errors in calculating Gibbs activation energy. The TST equation^18^ is exponentially dependent on this energy, so a 1 kcal mol^–1^ error in Gibbs activation energy can lead to a 5-fold error in the TST rate constant at 298 K, with a 2 kcal mol^–1^ can cause 29-fold error. The harmonic-oscillator approximation (HO) is typically used to calculate Gibbs activation energy and is reliable for high-frequency vibrations,^19^ but often inaccurate for low-frequency modes like hindered rotations (also known as torsions or internal rotations).^20−22^ In such cases, the hindered rotor (HR) model^23^ provides a more accurate estimation.

Both CVT and HR models, while valuable tools for calculating rate constants, present computational challenges. CVT requires identifying the optimal dividing surface to minimize recrossing, which involves computing the geometric and electronic properties of nonstationary points along the reaction pathway,^11^ increasing computational cost. Many theoreticians analyze only a few nonstationary points based on their expertise.^24−26^ On the other hand, implementing the HR model adds complexity by requiring detailed data for each rotating molecular fragment.^23^

To address these challenges, we developed an algorithm based on Toro-Labbe’s Reaction Force Analysis (RFA)^27^ to efficiently estimates CVT rate constants using a reduced set of nonstationary points along the reaction path, eliminating the need for empirical selection and reducing computational demands by ∼90%. *Eyringpy* also implements torsion treatments using Pitzer-Gwinn^28^ and Ayala-Schlegel^20^ one-dimensional models, with algorithms that simplify the process.^29^ While several codes exist for CVT (Table S1) and HR^23^ computations, to the best of our knowledge, *Eyringpy* is the only software that identifies the most relevant nonstationary points for accurate CVT rate constant estimation at low computational cost, applying RFA for this purpose.

### Canonical Variational Transition State Theory

In TST, the dividing surface is placed at the first-order saddle point on the PES, specifically at the transition state, assuming recrossing, which often leads to overestimated rate constants.^9−11,30^ CVT improves this by optimizing the dividing surface, minimizing the generalized TST rate constant, *k*_GT_, with respect to the reaction coordinate, *s*, as shown in eq 1.
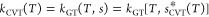
1Here, *k*_CVT_ is the CVT rate constant and *s* is the distance along the minimum-energy path (MEP) in isoinertial coordinates (e.g.*,* mass-weighted Cartesians or mass-scaled Cartesians). The MEP combines paths of steepest descent from the saddle point toward the reactants and products, with its origin at the saddle point (*s* = 0). On this path, *s* is positive toward the product side (*s* > 0) and negative toward the reactant side (*s* < 0). Any dividing surface intersecting the MEP at a specific *s* is considered a generalized transition state (GTS).^9,11,12,17,30,31^ The CVT transition state (TS_CVT_), located at *s*_CVT_^*^, minimizes *k*_GT_ at a given temperature, *T*.^12,17,31−33^ When the TS_CVT_ coincides with the saddle point, *k*_CVT_ converges to *k*_TST_.

*Eyringpy* uses the thermodynamic formulation of *k*_GT_, expressed in eq 2:
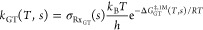
2where *k*_B_ is the Boltzmann constant, *h* is the Planck constant, *R* is the gas constant, and Δ*G*_GT_^‡,1M^ is the generalized Gibbs activation energy at standard concentration (c^o^ = 1 M). This aligns with the methodology employed by Galano and Alvarez-Idaboy for TST,^4^ allowing a more accurate determination of Δ*G*_GT_^‡,1M^. A critical aspect involves considering the equilibrium constant in terms of concentrations (*K*_c_GT__^‡^), defined by eq 3:
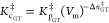
3
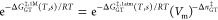
4Here, *K*_p_GT__^‡^ is the equilibrium constant in terms of partial pressures. *Eyringpy* calculates the generalized Gibbs activation energy at 1 atm, Δ*G*_GT_^‡,1 atm^, using the canonical partition function.^18^ The molar volume *V*_m_ is determined from the ideal gas law (*PV* = *nRT*), and Δ*n*_GT_^‡^ represents the difference in moles between the GTS and the reactants (0 for unimolecular and −1 for bimolecular reactions). Thus,
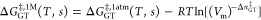
5
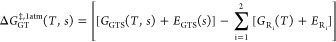
6where *G*_GTS_ and *E*_GTS_ are the Gibbs and the electronic energies of the GTS, and *G*_R_i__ and *E*_R_i__ are the Gibbs and the electronic energies of the *i*th reactant.

**Figure 1:**
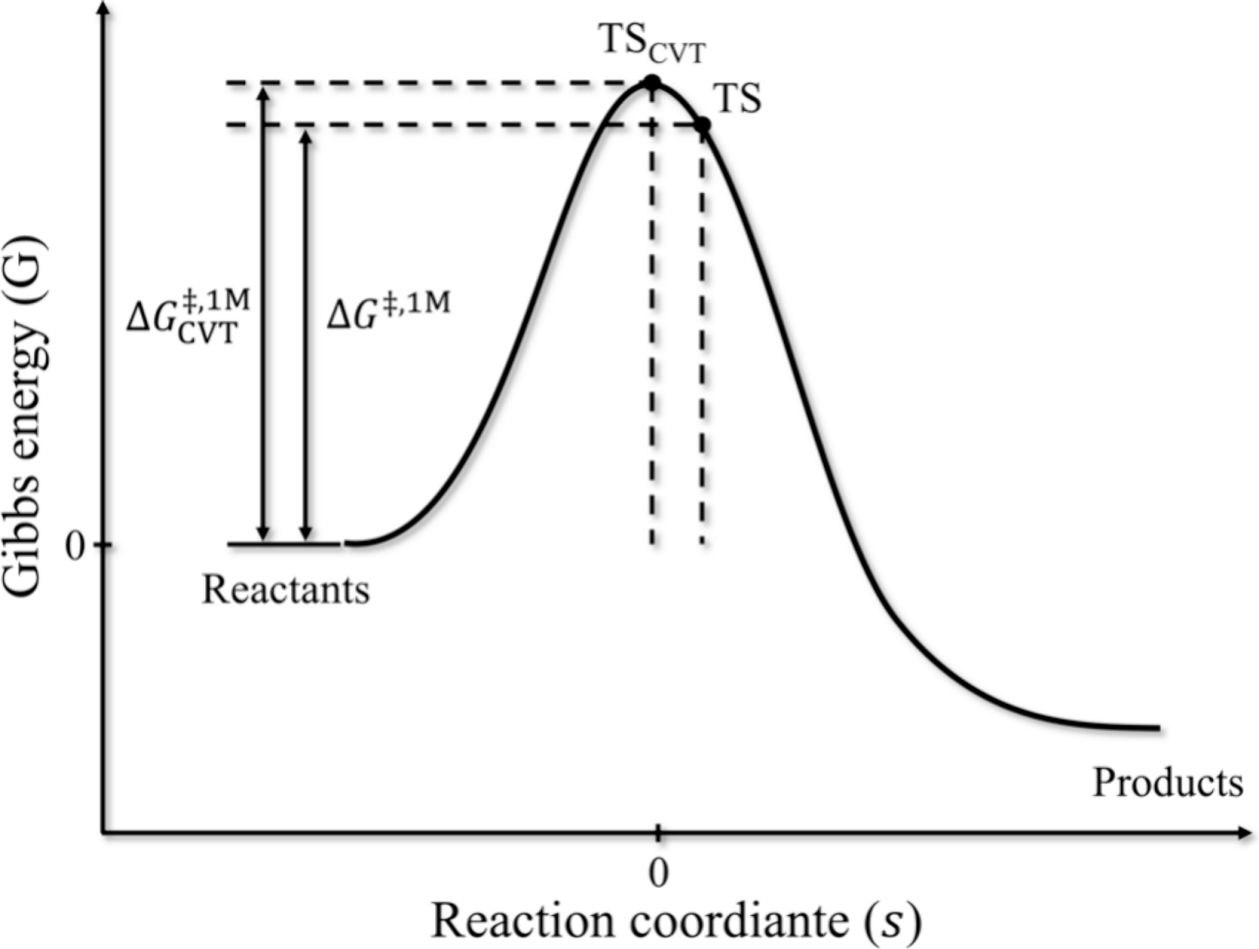
Profile of Gibbs energy along the reaction coordinate.

Minimize *k*_GT_ is equivalent to maximizing the generalized Gibbs activation energy with respect to *s* (eq 7).^9,11,12,17^ This process included the effects of zero-point energy (ZPE) and entropy, as shown in Figure 1.
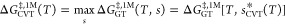
7

Typically, the reaction symmetry factor, σ_Rx_GT__(*s*), is calculated as the ratio between the product of the rotational symmetry numbers of the reactants, σ_i_^R^, and the rotational symmetry number of the GTS along the reaction coordinate σ_GTS_(*s*).
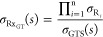
8where *n* represents the number of reactants.^11,34^ However, *Eyringpy* adopts a convention where σ_GTS_ is independent of *s*. In simpler terms, σ_GTS_ is determined as σ_TS_,^11,35,36^ following the same approach as in traditional TST.^18^

Calculating the reaction symmetry factor can become more complex in cases involving chiral molecules, multiple conformers, internal rotations, or symmetric reactions.^11,34^*Eyringpy* handles these challenges by systematically excluding rotational symmetry numbers from the canonical partition functions during the computation of generalized Gibbs activation energy.^18^

Consequently, the CVT rate constant in *Eyringpy* is determined by the following equation:
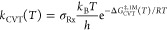
9

While CVT provides a more robust kinetic theory than TST, it demands a larger data set. Unlike TST, CVT needs geometric and electronic information for stationary points (reactants and TS) and a series of nonstationary points along the MEP, which is obtained by computing the intrinsic reaction coordinate (IRC) in isoinertial coordinates.^37,38^ A key parameter in IRC calculation is the step size; smaller step sizes are needed for curved reaction paths to ensure accuracy, whereas flatter paths can tolerate larger steps.^39^ An inaccurate step size may lead to misidentification of the variational TS on the MEP, resulting in inaccurate rate constants.

Traditionally, the selection of nonstationary points for CVT calculations relies on user expertise.^24−26^ However, *Eyringpy* incorporates an algorithm that uses the RFA scheme to objectively and accurately identify the most relevant points along the IRC. Further details on RFA can be found in the comprehensive reviews by Toro-Labbé.^27^

*Eyringpy* extracts the reaction coordinate and the electronic energy at each point of the IRC to build the MEP. The TS is placed at *s* = 0, with reactants at *s* < 0 and products at *s* > 0, using the energy of the reactants as the zero-point reference. The reaction force *F* is calculated as the negative gradient of the potential energy *V*(*s*) with respect to *s*,
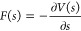
10

**Figure 2:**
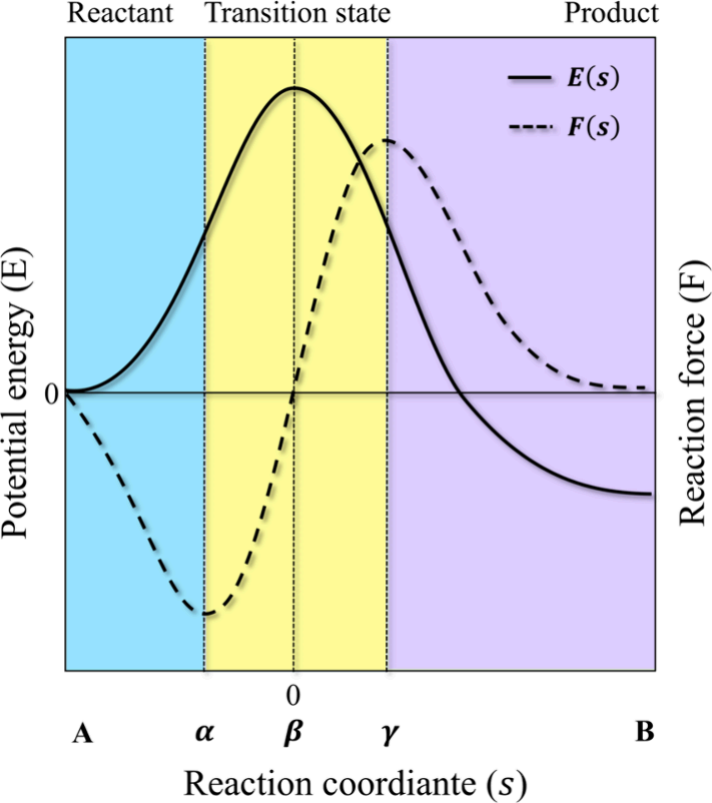
Potential energy (solid line) and reaction force (dashed line) along the reaction coordinate. Points α, β, and γ are the minimum, zero, and maximum of the reaction force.

The reaction force exhibits a minimum and a maximum at the inflection points of *V*(*s*), labeled α and γ, respectively, and is zero at the reactants, TS, and products (see Figure 2). These points define three zones along *s*:

1) Reactant zone (A → α): Characterized by structural changes in the reactants (bond stretching, angle bending, rotations, etc.).
2) Transition state zone (α → γ): Where primary electronic effects occur (bond breaking and formation).
3) Product zone (γ → B): Characterized by further structural changes leading to the products.

*Eyringpy* strategically selects nonstationary points within the TS zone, excluding the IRC point corresponding to the TS, as CVT requires the optimized structure of the TS. This includes the variational TS, which is crucial for accurately computing Δ*G*_CVT_^‡,1M^ at each temperature. This approach significantly reduces computational costs and provides a solid foundation for identifying key points along the MEP necessary for computing accurate CVT rate constants.

### Hindered Rotor Model

The methodology for determining thermodynamic properties is based on the canonical partition function
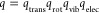
11

This function includes translational (*q*_trans_), rotational (*q*_rot_), and electronic (*q*_elec_) components, calculated using standard methods.^40^ However, the vibrational component (*q*_vib_) is challenging, particularly for low-frequency modes like hindered rotations. The harmonic oscillator (HO) approximation, though efficient for high-frequency vibrations,^19^ can introduce errors for low-frequency modes.^21,22^ To address this limitation, *Eyringpy* uses the hindered rotor model for a more accurate treatment of torsional modes.^23^

Our implementation decouples the total partition function into contributions from nontorsional and torsional modes (eqs 12–14).^41^ The HO approximation remains valid for nontorsional modes, while torsional modes are treated using the one-dimensional hindered rotor formulations from Pitzer and Gwinn (PG),^28^ and Ayala and Schlegel (AS)^20^:
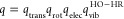
12
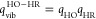
13
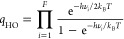
14Here, *q*_HO_ and *q*_HR_ are the partition functions for the quantum harmonic oscillator and hindered rotor, respectively; ν_i_ denotes the vibrational frequency of the *i*th nontorsional mode, and F is the number of nontorsional modes.

### Model of Pitzer and Gwinn

The PG method refines the classical partition function (*q*_class_) by incorporating the ratio of the quantum HO partition function (*q*_HO_) to its classical counterpart (*q*_CHO_), eq 15.
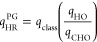
15
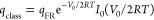
16
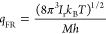
17
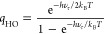
18
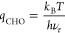
19where *q*_FR_ is the free rotor partition function, *I*_0_ is the modified Bessel function of the first type of zeroth-order, *I*_r_ is the reduced moment of inertia, *M* is the total number of minima along the torsional coordinate within the 0–2π range, ν_τ_ is the torsional frequency, and *V*_0_ is the torsional barrier,^28^ determined using the formula by Ayala and Schlegel (eq 20).^20^
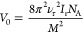
20where *N*_A_ is the Avogadro constant.

The PG scheme provides accurate results for molecules with a single torsion, particularly when the potential is represented by *V*_0_(1 – cos *M*φ)/2.^28^ It also performs well for multiple, well-separated rotors, as detailed in eq 21(20):
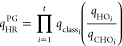
21where *t* is the number of torsional modes.

Using the PG model, we derived formulas for the thermodynamic properties of HRs by incorporating eq 15 into the standard statistical thermodynamics equations (see Tables 1 and 2).^40^ To our knowledge, only the equations for *H*_class_^PG^ and *G*_class_^PG^ have been previously documented.^28^

**Table 1:** Internal Energy and Entropy According to the Hindered Rotor Model of Pitzer and Gwinn, Per Mole of Substance^a^

	internal energy	entropy
free rotor	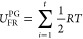	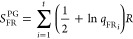
classical harmonic oscillator	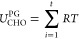	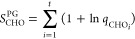
quantum harmonic oscillator	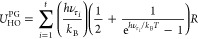	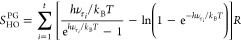
classical	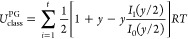	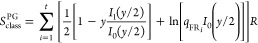
total	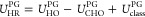	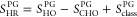

a *y* = *V*_0_/*RT*. *I*_0_ is the modified Bessel function of the first kind of zeroth order. *I*_1_ is the modified Bessel function of the first kind of first order.

**Table 2:** Enthalpy and Gibbs Energy According to the Hindered Rotor Model of Pitzer and Gwinn, Per Mole of Substance^a^

	enthalpy	Gibbs energy
free rotor	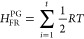	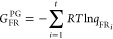
classical harmonic oscillator	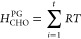	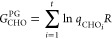
quantum harmonic oscillator	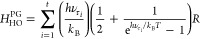	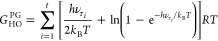
classical	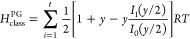	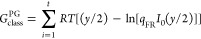
total	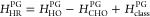	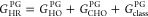

a *y* = *V*_0_/*RT*. *I*_0_ is the modified Bessel function of the first kind of zeroth order. *I*_1_ is the modified Bessel function of the first kind of first order.

### Model of Ayala and Schlegel

Ayala and Schlegel (AS) improved the PG model to account for low torsional barriers and high temperatures by introducing an adjustment factor (eq 22), based on accurate tabulated values from PG.^28^ The AS approximation is formulated as
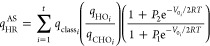
22Here, *P*_1_ and *P*_2_ are fifth-order polynomial functions of *x* = 1/*q*_FR_ and *y* = *V*_0_/*RT*.^20^

We have derived the thermodynamic property equations for HR using the AS scheme, which are unavailable in current literature (see Table 3). The polynomial expressions for P_3_ and P_4_ were computed using the *SymPy Python* library for precise calculations (see Table S2), with a default precision of 15 digits.

**Table 3:** Internal Energy, Entropy, Enthalpy, and Gibbs Energy According to the Hindered Rotor Model of Ayala and Schlegel, Per Mole of Substance^a^

internal energy	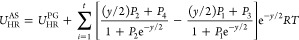
entropy	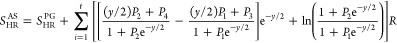
enthalpy	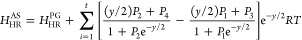
Gibbs energy	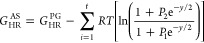

a and  are fifth-order polynomial functions.

### Tests

To show these functionalities in *Eyringpy*, we applied it to two gas-phase reactions. The first between formic acid and the hydroxyl radical, highlights the importance of CVT in capturing recrossing effect, which are critical in low-barrier reactions at higher temperatures. The second, between methane with the hydroxyl radical, shows the effectiveness of *Eyringpy* in estimating rate constants for torsional motions, using the HR models developed by Pitzer-Gwinn^28^ and Ayala-Schlegel.^20^

### Hydrogen Abstraction from Formic Acid by Hydroxyl Radical

Full geometry optimizations and frequency analysis were performed at the M05-2X^42^/6-311++G(d,p) level using *Gaussian 09* Rev D.01.^43^ Local minima and TSs were identified by their number of imaginary frequencies (0 and 1, respectively). Unrestricted calculations were used for open-shell systems. To confirm the TSs connect reactants and products, the IRC^37,38^ was computed using the local quadratic approximation (LQA) for the predictor step.^44,45^ The MEP was traced by following the IRC downhill from the TS to the reactants and products. Mass-weighted internal coordinates were used without reorientation or symmetry adjustments. For each TS, one hundred points were computed on both sides of the path with a step size of 0.05 amu^1/2^ bohr.

We focused on two pathways: (I) hydrogen abstraction from the carboxyl group and (II) hydrogen abstraction from the formyl group. Consistent with Anglada’s results,^46^ we excluded addition pathways to the CO group. Geometrical parameters of reactants and products (Figure S1) match previously reported theoretical^46,47^ and experimental^48−50^ data. Both pathways follow a two-step mechanism: (1) barrierless formation of a reactant complex (RC) in equilibrium with the reactants, followed by (2) irreversible formation of the products.
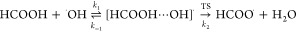
I
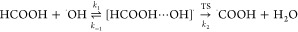
II

#### H-Abstraction from COOH

**Figure 3:**
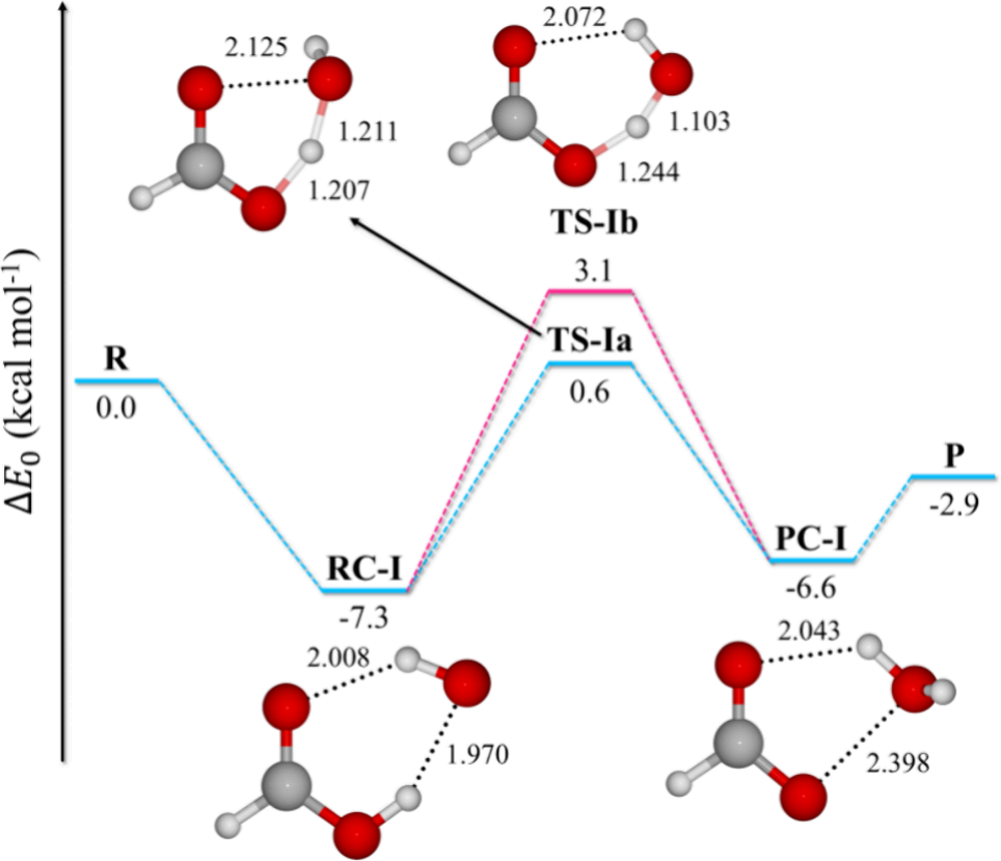
Electronic energy + ZPE profiles (in kcal mol^–1^) of the carboxylic H-abstraction paths (Ia and Ib) at the M05-2*X*/6-311++G(d,p) level. Bond lengths in Å.

Our study identified two distinct H-abstraction pathways (Figure 3). Both originate from the same reactant complex (RC-I) and converge at an identical product complex (PC-I). Path Ia follows a proton-coupled electron transfer (PCET) mechanism, as proposed by Anglada,^46^ while path Ib proceeds via hydrogen atom transfer (HAT). Based on our calculations, path Ia is more favorable.

#### H-Abstraction from CHO

**Figure 4:**
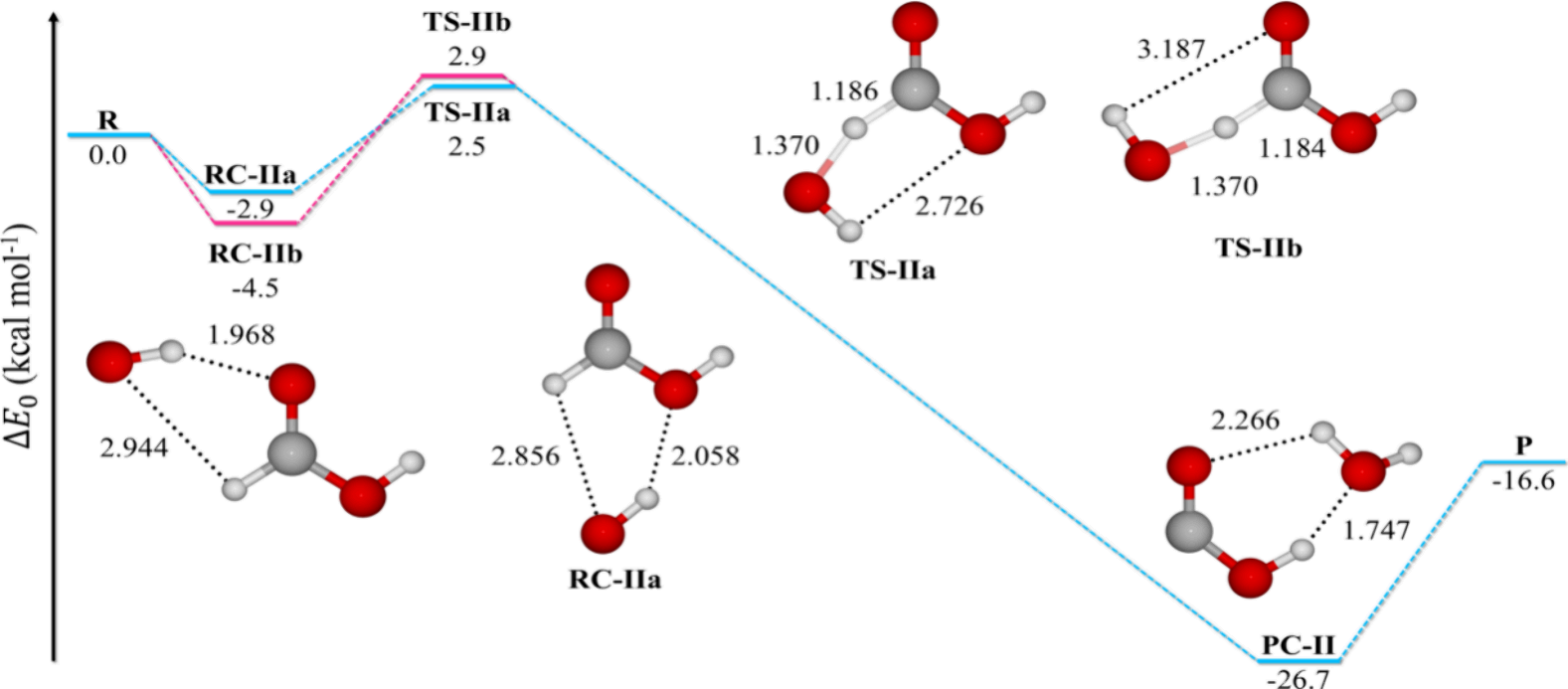
Electronic energy + ZPE profiles (in kcal mol^–1^) of the formyl H-abstraction pathways (IIa and IIb) at the M05-2*X*/6-311++G(d,p) level. Bond lengths are in Angstrom.

We identified two distinct TSs, TS-IIa and TS-IIb, differing in the orientation of the OH radical relative to formic acid (Figure 4). In TS-IIa, OH approaches the hydroxyl oxygen, while in TS-IIb, it targets the carbonyl oxygen. Despite these geometric differences, the *E*_0_ values of both TSs differ by only 0.4 kcal mol^–1^. Previous studies treated the rate constants of these pathways cumulatively. However, Iuga et al.^51^ noted a minimal barrier between TS-IIa and TS-IIb due to the nearly free rotation around the O···H bond with the transferred hydrogen atom. They suggested choosing the pathway with the higher individual rate constant for greater accuracy, rather than summing the rate constants.

### Reaction Force Analysis

**Figure 5:**
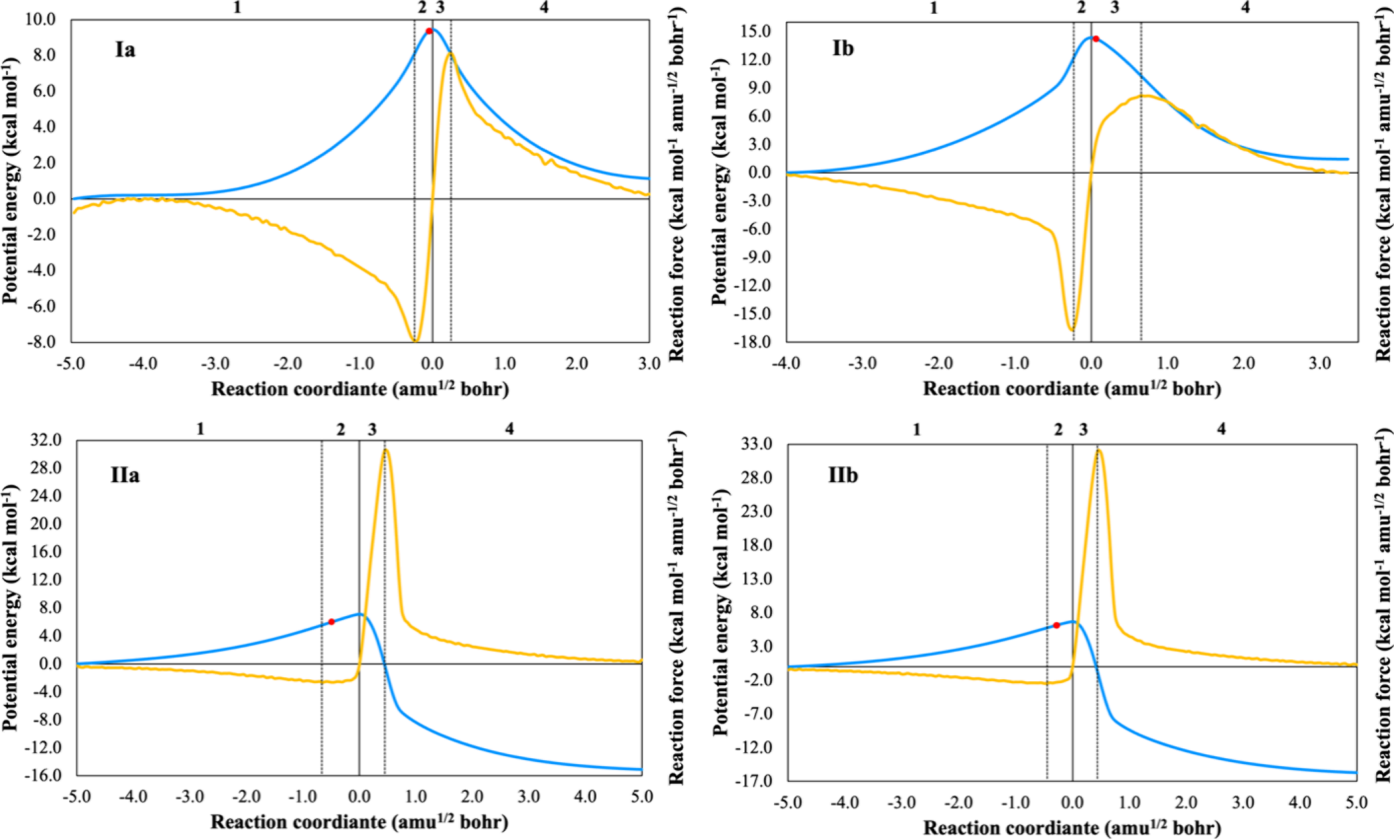
Potential energy (blue line) and reaction force (orange line) profiles for the hydrogen abstractions paths from CH_4_ by ·OH. *F*(*s*) profile divides the energy curves into four regions: reactant (1), transition state (2 and 3), and product (4). Red dot marks the variational TS at 298.15 K.

Accurate calculation of CVT rate constants depends on the proper selection of nonstationary points along the MEP.^24,25^ We used the RFA scheme to identify the most important points. Figure 5 shows the *V*(*s*) and *F*(*s*) profiles of the different reaction paths. Among the regions defined by *F*(*s*), the TS zone is the most significant for CVT. Path Ia has a sharp *V*(*s*) curve, while paths IIa and IIb displays flatter curves. In contrast, path Ib shows both sharp and a flat section, affecting the number of nonstationary points in the TS zone (see Table 4), which is crucial for CVT.

**Table 4:** Nonstationary Points Along the IRC, in the TS Zone, and for CVT (%)

path	Ia	Ib	IIa	IIb
IRC points	168	156	201	201
TS zone points	10	18	22	18
*P*_CVT_ (%)	6.0	11.5	10.9	9.0

We also calculated the percentage of nonstationary points required for CVT as *P*_CVT_ (%*T*) = (TS zone points/IRC points) × 100. Remarkably, our algorithm requires only 6.0–11.5% of the IRC points, significantly reducing computational costs (see Table 4). Additionally, it accurately locates the variational TS. Yielding precise CVT rate constants. For paths Ia and Ib, the variational TS is near the saddle point (−0.05 and 0.05 amu^1/2^ bohr, respectively), while for paths IIa and IIb, it is closer to the reactant zone (−0.5 and −0.3 amu^1/2^ bohr, respectively).

### Rate Constants and Branching Ratios

We calculated the overall rate constant (*k*_overall_) for the reaction between formic acid with hydroxyl radical by summing the rate constants of each individual pathway:
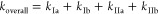
23

**Figure 6:**
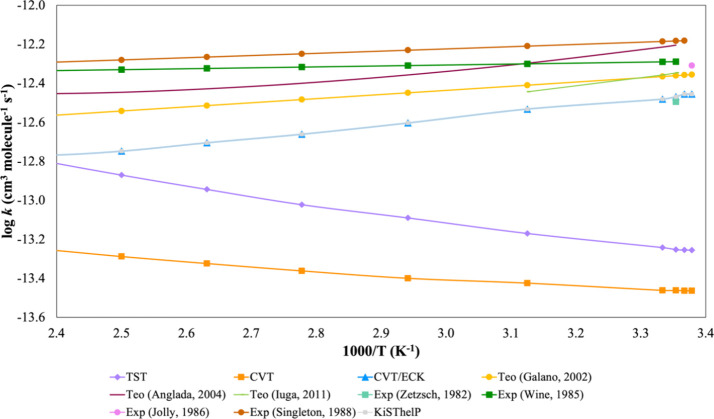
Overall rate constants (in cm^3^ molecule^–1^ s^–1^) for the hydrogen abstraction from HCOOH by ·OH in the gas phase within the 296–440 K temperature range.

Kinetic computations were performed over the 296–440 K range, where most experimental data is available. Table 5 and Figure 6 summarize the global rate constants, which agree with reported theoretical^46,47,51^ and experimental^52−55^ results, with a maximum deviation of 2.6-fold from experiment. We also included TST rate constants for comparison, emphasizing the need for CVT to achieve accurate results. As expected, TST tends to overestimate the experimental values by up to 8.8-fold, while CVT significantly improves the accuracy, although the extend for improvement varies with temperature. Table S3 shows the recrossing factor (*k*_TST_/*k*_CVT_), quantifying the variational effect. This effect is negligible in paths Ia and Ib but reaches 2 orders of magnitude in paths IIa and IIb.

**Table 5:** Overall Rate Constants (in cm^3^ molecule^–1^ s^–1^) of the Gas Phase Hydrogen Abstraction from Formic Acid by ·OH in the 296–440 K Range^a^^,^^b^^,^^c^^,^^d^^,^^e^

*T*	TST	CVT	CVT/ECK	theo^47^	theo^46^	theo^51^	exp^55^	exp^54^	exp^52^	exp^53^	*KiSThelP*(56)
296	5.6 × 10^–14^	3.4 × 10^–14^	3.5 × 10^–13^	4.4 × 10^–13^					4.9 × 10^–13^		3.4 × 10^–14^
297	5.6 × 10^–14^	3.4 × 10^–14^	3.5 × 10^–13^	4.4 × 10^–13^						6.6 × 10^–13^	3.4 × 10^–14^
**298**	**5.6 × 10^–14^**	**3.4 × 10^–14^**	**3.4 × 10^–13^**	**4.4 × 10^–13^**	**6.2 × 10^–13^**	**4.5 × 10^–13^**	3.2 × 10^–13^	**5.1 × 10^–13^**		**6.6 × 10^–13^**	**3.4 × 10^–13^**
300	5.7 × 10^–14^	3.4 × 10^–14^	3.3 × 10^–13^	4.3 × 10^–13^	6.1 × 10^–13^	4.4 × 10^–13^		5.1 × 10^–13^		6.5 × 10^–13^	3.3 × 10^–13^
320	6.8 × 10^–14^	3.8 × 10^–14^	2.9 × 10^–13^	3.9 × 10^–13^	5.0 × 10^–13^	3.6 × 10^–13^		5.0 × 10^–13^		6.2 × 10^–13^	2.9 × 10^–13^
340	8.1 × 10^–14^	4.0 × 10^–14^	2.5 × 10^–13^	3.6 × 10^–13^	4.4 × 10^–13^			4.9 × 10^–13^		5.9 × 10^–13^	2.5 × 10^–13^
360	9.5 × 10^–14^	4.3 × 10^–14^	2.2 × 10^–13^	3.3 × 10^–13^	4.0 × 10^–13^			4.8 × 10^–13^		5.6 × 10^–13^	2.2 × 10^–13^
380	1.1 × 10^–13^	4.7 × 10^–14^	2.0 × 10^–13^	3.1 × 10^–13^	3.7 × 10^–13^			4.7 × 10^–13^		5.4 × 10^–13^	2.0 × 10^–13^
400	1.3 × 10^–13^	5.1 × 10^–14^	1.8 × 10^–13^	2.9 × 10^–13^	3.6 × 10^–13^			4.7 × 10^–13^		5.2 × 10^–13^	1.8 × 10^–13^
420	1.6 × 10^–13^	5.6 × 10^–14^	1.7 × 10^–13^	2.7 × 10^–13^	3.5 × 10^–13^			4.6 × 10^–13^		5.1 × 10^–13^	1.7 × 10^–13^
440	1.9 × 10^–13^	6.0 × 10^–14^	1.7 × 10^–13^	2.6 × 10^–13^	3.5 × 10^–13^					4.9 × 10^–13^	1.7 × 10^–13^

a MP2/6-311++G(2d,2p)//MP2/6-311++G(2d,2p).^47^

b CCSD(T)/aug-cc-pVTZ//QCISD/6-311+G/2df,2p).^46^

c M05-2*X*/6-311G++(d,p).^51^

d Flash photolysis-resonance fluorescence.^54,55^

e Pulsed laser photolysis-resonance absorption.^52,53^

Table S4 presents the branching ratios Γ, showing each pathway’s contribution to the global rate constant. Pathway Ia dominates, contributing 97.3% at 296 K to 75.1% at 440 K, while pathway IIb has the smallest contribution (0.1–0.8%). Pathway Ia dominance decreases slightly with increasing temperature, while pathway Ib contributes 2.3% at 296 K and Γ_IIb_ increases to 19.1% at 440 K.

The CVT rate constants obtained using *Eyringpy* align closely with those from *KiSThelP*.^56^*Eyringpy*’s user-friendly interface requires only a simple input file containing geometric and electronic data of the stationary points from the output files, along with a few nonstationary points, significantly improving computational efficiency. In this case, it took just 1.8 s to calculate the CVT rate constants using *Eyringpy*.

### Hydrogen Abstraction from Methane by Hydroxyl Radical

All geometries were fully optimized at the BhandHLYP/6-311G(d,p) level. Harmonic vibrational frequency analysis confirmed the nature of each stationary point. To improve accuracy, single-point energies were calculated at the CCSD(T)/6-311G(d,p) level, with Gibbs free energies incorporating thermal contributions from BhandHLYP/6-311G(d,p). The IRC was performed using the LQA approximation,^44,45^ to verify that the transition states connect the correct reactants and products. Parameters for hindered rotors, including torsional frequency, symmetry, and reduced moment of inertia, were determined using Ayala and Schlegel’s algorithm.^20^ The torsional potential was scanned in 20° increments from 0 to 360°. All computations were carried out using *Gaussian 09* Rev. D.01.^43^

Thermal rate constants were computed via TST in *Eyringpy*, with tunneling factors based on a one-dimensional Eckart potential energy barrier.^57^ For low-frequency vibrational modes corresponding to internal rotations, *Eyringpy* used the one-dimensional models of Pitzer–Gwinn^28^ and Ayala–Schlegel^20^ to calculate partition functions and thermodynamic properties.

**Figure 7:**
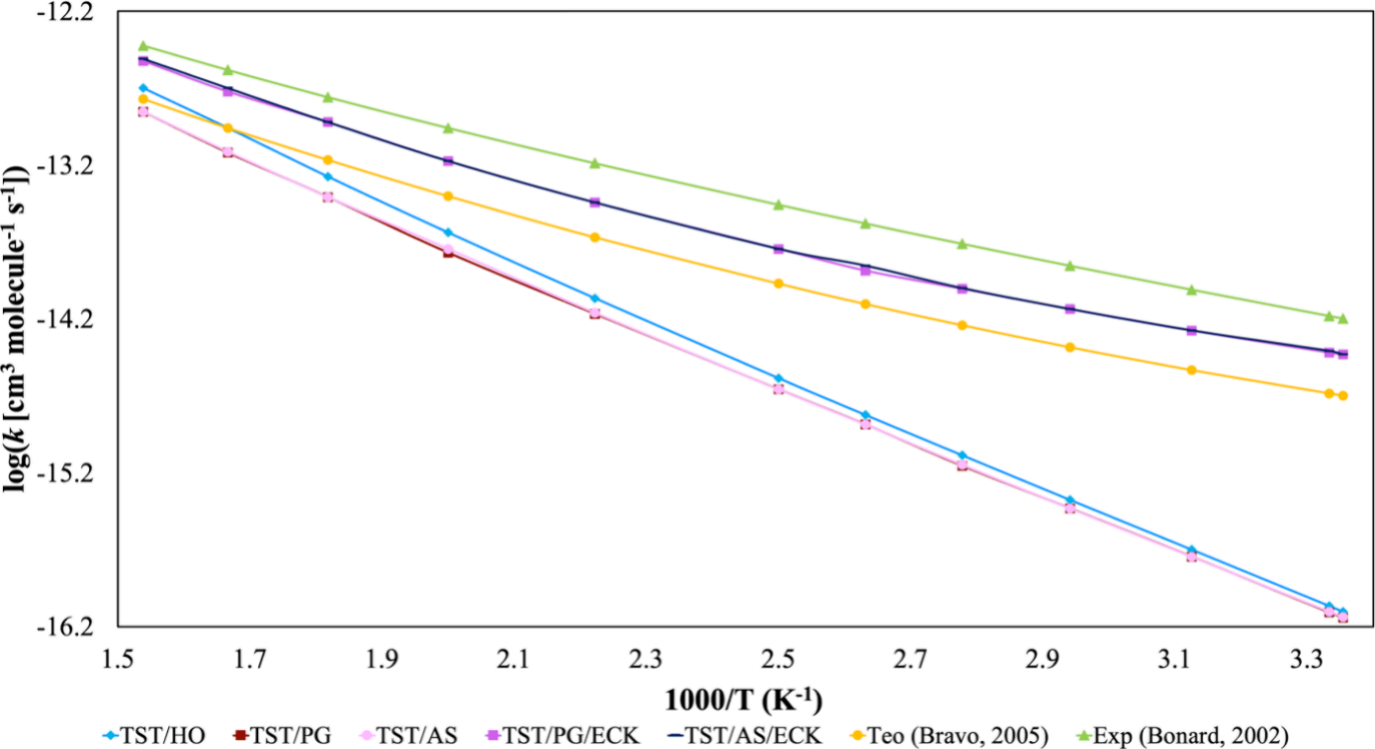
Overall rate constants (in cm^3^ molecule^–1^ s^–1^) for hydrogen abstraction from CH_4_ by ·OH in the gas phase within the 298–650 K temperature range.

Table 6 and Figure 7 summarize the rate constants across 298–650 K. Vibrational modes were treated as harmonic oscillators, except for the hindered rotation of the −CH_3_ group in the TS, which had a torsional frequency of 54 cm^–1^ and a barrier of 0.4 kcal mol^–1^. Neglecting the tunneling underestimates TST rate constants by up to 2 orders of magnitude, highlighting the importance of tunneling corrections, especially in light particle transfers like hydrogen. In terms of vibration treatment, using the HR approximation improved TST agreement with experimental data, though the improvement was modest due to the low barrier (less than *RT*) for −CH_3_ rotation. The *k*_TST_^HO^/*k*_TST_^HR^ ratio ranged from 1.1 at 298 K to 1.4 at 650 K for both the PG and AS models.

**Table 6:** Rate Constants (in cm^3^ molecule^–1^ s^–1^) as a Function of the Temperature of the Hydrogen Abstraction from CH_4_ by ·OH in the Gas Phase

*T* (K)	TST/HO	TST/PG	TST/AS	TST/PG/ECK	TST/AS/ECK	theo^a^	exp^b^	*TheRate*(24)
**298**	**7.90 × 10^–17^**	**7.20 × 10^–17^**	**7.30 × 10^–17^**	**3.70 × 10^–15^**	**3.70 × 10^–15^**	**2.00 × 10^–15^**	**6.33 × 10^–15^**	**3.70 × 10^–15^**
300	8.60 × 10^–17^	7.80 × 10^–17^	7.90 × 10^–17^	3.80 × 10^–15^	3.90 × 10^–15^	2.07 × 10^–15^	6.58 × 10^–15^	3.90 × 10^–15^
320	2.00 × 10^–16^	1.80 × 10^–16^	1.80 × 10^–16^	5.30 × 10^–15^	5.30 × 10^–15^	2.93 × 10^–15^	9.76 × 10^–15^	5.30 × 10^–15^
340	4.20 × 10^–16^	3.70 × 10^–16^	3.70 × 10^–16^	7.30 × 10^–15^	7.30 × 10^–15^	4.12 × 10^–15^	1.40 × 10^–14^	7.30 × 10^–15^
360	8.20 × 10^–16^	7.00 × 10^–16^	7.10 × 10^–16^	9.90 × 10^–15^	1.00 × 10^–14^	5.73 × 10^–15^	1.94 × 10^–14^	1.00 × 10^–14^
380	1.50 × 10^–15^	1.30 × 10^–15^	1.30 × 10^–15^	1.30 × 10^–14^	1.40 × 10^–14^	7.87 × 10^–15^	2.63 × 10^–14^	1.40 × 10^–14^
400	2.60 × 10^–15^	2.20 × 10^–15^	2.20 × 10^–15^	1.80 × 10^–14^	1.80 × 10^–14^	1.07 × 10^–14^	3.48 × 10^–14^	1.80 × 10^–14^
450	8.60 × 10^–15^	6.80 × 10^–15^	6.90 × 10^–15^	3.60 × 10^–14^	3.60 × 10^–14^	2.14 × 10^–14^	6.47 × 10^–14^	3.60 × 10^–14^
500	2.30 × 10^–14^	1.70 × 10^–14^	1.80 × 10^–14^	6.70 × 10^–14^	6.70 × 10^–14^	3.96 × 10^–14^	1.10 × 10^–13^	6.70 × 10^–14^
550	5.30 × 10^–14^	3.90 × 10^–14^	3.90 × 10^–14^	1.20 × 10^–13^	1.20 × 10^–13^	6.81 × 10^–14^	1.75 × 10^–13^	1.20 × 10^–13^
600	1.10 × 10^–13^	7.60 × 10^–14^	7.70 × 10^–14^	1.90 × 10^–13^	2.00 × 10^–13^	1.10 × 10^–13^	2.62 × 10^–13^	2.00 × 10^–13^
650	2.00 × 10^–13^	1.40 × 10^–13^	1.40 × 10^–13^	3.00 × 10^–13^	3.10 × 10^–13^	1.70 × 10^–13^	3.77 × 10^–13^	3.10 × 10^–13^

a Theoretical rate constants were calculated at level: CCSD(T)/6-311G(d,p)//BHandHLYP/6-311G(d,p).^58^

b Experimental rate constants were measured with the method of pulse laser photolysis/laser induced fluorescence.^59^

### How Do We Compute CVT Rate Constants in *Eyringpy*?

*Eyringpy* streamlines CVT rate constant calculations. Here’s an overview.

### Collecting the Input Data

For a CVT calculation, *Eyringpy* requires electronic structure data for all stationary points (reactants, products, TS, and reactant and/or product complexes) and selected nonstationary points along the MEP. *Eyringpy* parses these data from *Gaussian* output files (*.out or *.log).

### Writing the Input File for the Frequency Analysis of Each Nonstationary Point

*Eyringpy* automates the identification of critical nonstationary points along the IRC, simplifying frequency calculations. To activate this feature, users must include the ‘METHOD CVT’ keyword in the *Eyringpy* input file and specify IRC output filename(s) using the keyword ‘IRC’. Instructions for the *Gaussian* frequency analysis are provided via ‘PATHPOINTS_INP.’ For more details, refer to the *Eyringpy* user’s manual.

### Creating the *Eyringpy* Input File

**Figure 8:**
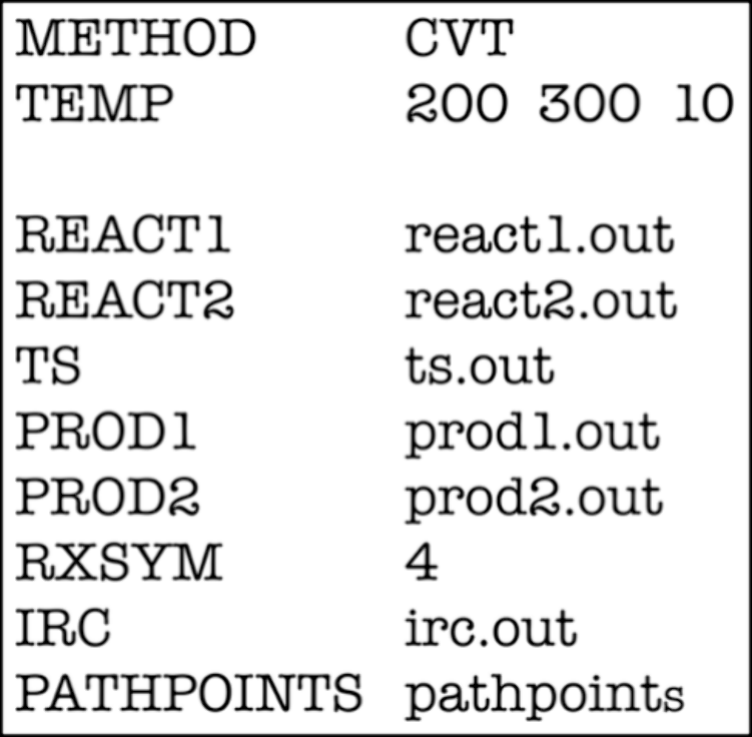
Example of an *Eyringpy* input file to compute CVT rate constants.

*Eyringpy* uses a straightforward input file format (*.eif) for CVT calculations (Figure 8). The file consists of key-value pairs such as ‘key name value’. The four main keyword categories for CVT include:

1. ‘METHOD’ to initiate the CVT calculation.
2. Keywords specifying output filenames for stationary points: ‘REACT1’, ‘REACT2’, ‘TS’, ‘PROD1’, ‘PROD2’.
3. ‘RXSYM’ to define reaction symmetry (if applicable)
4. ‘IRC’ for IRC output filenames and ‘PATHPOINTS’ for the directory with nonstationary points.

### How Do We Compute HR Rate Constants in *Eyringpy*?

#### Obtaining the Hindered Rotor Input Parameters

The HR model requires parameters like the reduced moment of inertia *I*_r_, torsional frequency ν_τ_, torsional barrier *V*_0_, and torsional symmetry number σ_τ_ (see eqs 15–22 and Tables 1–3). *Eyringpy* automatically computes *V*_0_ using eq 20. However, the remaining parameters can be provided in two ways:

1) Use *Gaussian* (09 or 16) output files containing the HR analysis.
2) Manually input the parameters using the keywords such as RED_MOM_INERT_*x*, TORFREQ_*x*, and TORNSYM_*x*, where *x* refers to the species in the reaction (R*n*, RC, TS, PC, or P*n*), and *n* ranges from 1 to 2 depending on the molecularity of the reaction. For available software to calculate HR parameters, see Dzib and Merino’s review.^23^

### Creating the *Eyringpy* Input File

**Figure 9:**
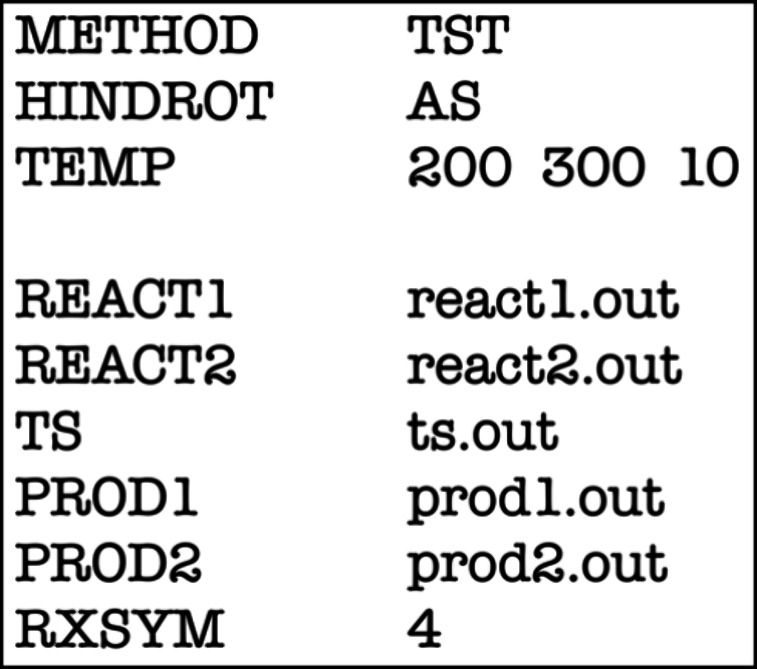
Example of an *Eyringpy* input file to compute HR rate constants.

Figure 9 shows an example of *Eyringpy* input file (*.eif) for HR rate constant computations. The “HINDROT” keyword specifies the HR model (Pitzer–Gwinn, PG, and Ayala–Schlegel, AS). Other HR-related keywords are described previously.

## Summary and Outlook

This new release of *Eyringpy* introduces two key functionalities for computing more accurate rate constants. First, it incorporates canonical variational transition state theory to address the recrossing effect, which is crucial for reactions with low activation energies and at high temperatures. *Eyringpy* efficiently locates relevant points along the reaction path for CVT using an algorithm based on reaction force analysis. Second, it integrates one-dimensional hindered rotor models from Pitzer-Gwinn and Ayala-Schlegel, enabling the calculation of reaction and activation energies for systems with internal rotations. True to *Eyringpy*’s philosophy, these modules require minimal input data while delivering reliable results. *Eyringpy* remains user-friendly and accessible, distributed as stand-alone, multiplatform software with effortless installation and immediate functionality. Download *Eyringpy* for free at https://www.theochemmerida.org/eyringpy.

## Supporting Information Available

Geometries of the reactants (R) and products (P) for hydrogen abstraction from formic acid by the hydroxyl radical, optimized at the M05-2*X*/6-311++G(d,p) level; polynomials for the Ayala and Schlegel hindered rotor model; recrossing factors (ratio *k*_TST_/*k*_CVT_) for gas-phase hydrogen abstraction from formic acid by ·OH in the 298.15–440 K range; and branching ratios, in percentage (PDF)
