# Supplementary material for: Enhancing Eyringpy: Accurate Rate Constants with Canonical Variational Transition State Theory and the Hindered Rotor Model
Source: J Chem Theory Comput. 2024 Nov 7;20(22):9999–10009. doi: 10.1021/acs.jctc.4c00926 (PMC11603600; doi:10.1021/acs.jctc.4c00926)
Supplement: Supplementary file 1 — ct4c00926_si_001.pdf [file ct4c00926_si_001.pdf]

Supporting Information

Enhancing *Eyringpy*: Accurate Rate Constants with Canonical Variational Transition State Theory and the Hindered Rotor Model

Eugenia Dzib,[1,*] Alan Quintal,[1] and Gabriel Merino.[1,*]

[1] Departamento de Física Aplicada, Centro de Investigación y de Estudios Avanzados, Km. 6 Antigua carretera a Progreso Apdo. Postal 73, Cordemex 97310, Mérida, México.

e-mail: eugenia.dzib@cinvestav.mx

gmerino@cinvestav.mx

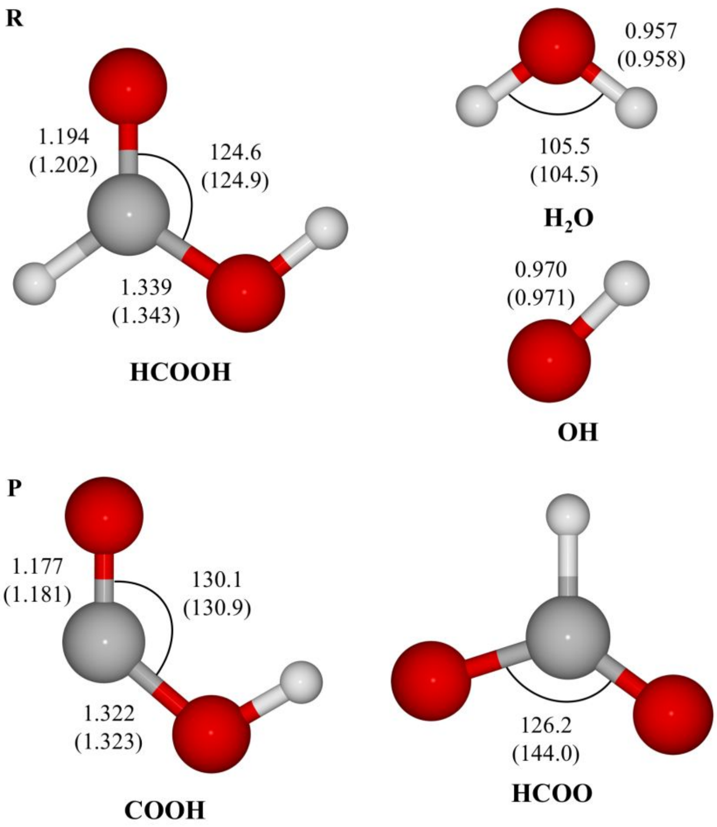

Figure S1. Geometries of the reactants (R) and products (P) of the hydrogen abstraction from formic acid by hydroxyl radical optimized at the M05-2X/6-311++G(d,p) level. Experimental values are between parentheses.[1-3] Bond lengths are in Angstrom and angles are in degrees.

Table S1. Software to compute rate constants with canonical variational transition state theory.

Software	Kinetic theory	Molecularity	Phase	Language	Gui	Operating system	Input data	Version
Eyringpy[4]	TST<br>CVT<br>MT<br>CKT	Uni<br>Bi	gas<br>solution	*Python*		W, M, L	Automatic	Binary
TheRate[5]	TST<br>CVT	Uni<br>Bi	gas	*Java*	*	W, M, L	Manual	App
KiSThelP[6]	TST<br>CVT<br>RRKM	Uni<br>Bi	gas	*Java*	*	W, M, L	Automatic<br>Manual	App
Polyrate[7-9]	TST<br>CVT<br>μVT<br>RRKM	Uni<br>Bi	gas<br>solid<br>gas-solid	*Fortran*		W, M, L	Automatic<br>Manual	Source
Pilgrim[10]	TST<br>CVT	Uni<br>Bi	gas	*Python*		M, L	Automatic<br>Manual	Source
TUMME[11]	TST<br>CVT<br>RRKM<br>ILT	Uni<br>Bi	gas	*Python,*<br>*C++*		L	Automatic<br>Manual	Source

[a]Not available, Uni: Unimolecular, Bi: Bimolecular, TST: Transition state theory, CVT: Canonical variational transition state theory, μVT: Microcanonical variational transition state theory, RRKM: Rice-Ramsperger-Kassel-Marcus, ME: Master equation, MT: Marcus theory, CKT: Collins-Kimball theory, ILT: Inverse Laplace transform, W: Windows, M: MacOS, and L: Linux.

Table S2. Polynomials for the hindered rotor model of Ayala and Schlegel.

$$P_1 = 0.003235x - 0.026252x^2 + 0.110460x^3 - 0.203340x^4 + 0.130633x^5 - 0.010112y^{\frac{1}{2}} + 0.650122xy^{\frac{1}{2}} + \\ - 0.364852y + 0.913073xy - 0.021116x^2y - 0.092086x^3y - 0.415689y^{\frac{3}{2}} \\ - 1.128961xy^{\frac{3}{2}} + 0.223009x^2y^{\frac{3}{2}} + 0.421344y^2 + 0.505139xy^2 - 0.215088x^2y^{\frac{5}{2}}$$

$$P_2 = 0.067113x + 0.772485x^2 - 3.0674131x^3 + 4.595051x^4 - 2.101341x^5 + 0.015800y^{\frac{1}{2}} \\ + 0.102119xy^{\frac{1}{2}} - 0.555270x^2y^{\frac{1}{2}} - 1.125261x^3y^{\frac{1}{2}} + 0.071884x^4y^{\frac{1}{2}} - 0.397330y \\ + 2.284956xy + 0.850046x^2y - 0.174240x^3y - 0.451875y^{\frac{3}{2}} - xy^{\frac{3}{2}} - 2.136226xy^{\frac{3}{2}} \\ + 0.303469x^2y^{\frac{3}{2}} + 0.470837y^2 + 0.675898xy^2$$

$$P_3 = -0.3265825x^5 + 0.035725x^4y^{\frac{1}{2}} + 0.406668x^4 - 0.177614x^3y^{\frac{1}{2}} + 0.230215x^3y - 0.16569x^3 \\ - 0.100668x^2y^{\frac{1}{2}} + 0.042232x^2y - 0.5825225x^2y^{\frac{3}{2}} + 0.026252x^2 - 0.650122xy^{\frac{1}{2}} \\ + 1.2628475xy^2 - 1.3696095xy + 2.257922xy^{\frac{3}{2}} - 0.0016175x + 0.005056y^{\frac{1}{2}} \\ - 0.842688y^2 + 0.364852y + 0.6235335y^{\frac{3}{2}} + 0.53772y^{\frac{5}{2}}$$

$$P_4 = 5.2533525x^5 - 0.17971x^4y^{\frac{1}{2}} - 9.190102x^4 + 2.250522x^3y^{\frac{1}{2}} + 0.4356x^3y + 4.60111965x^3 \\ + 0.832905x^2y^{\frac{1}{2}} - 1.700092x^2y - 0.7586725x^2y^{\frac{3}{2}} - 0772485x^2 - 0.102119xy^{\frac{1}{2}} \\ - 1.689745xy^2 - 3.427434xy + 4.272452xy^{\frac{1}{2}} - 0.0335565x - 0.0079y^{\frac{1}{2}} - 0.941674y^2 \\ + 0.39733y + 0.6778125y^{\frac{3}{2}} + 0.56577175y^{\frac{5}{2}}$$

Table S3. Recrossing factors (ratio k_{TST}/k_{CVT}) of the gas phase hydrogen abstraction from formic acid by ·OH in the 298.15-440 K range.

T (K)	Ia	Ib	IIa	IIb	Overall
296.0	1.09	1.15	9.17	49.41	1.62
297.0	1.09	1.13	9.17	47.22	1.62
**298.15**	**1.09**	**1.11**	**8.46**	**48.33**	**1.62**
300.00	1.09	1.20	9.23	47.37	1.66
320.00	1.09	1.14	8.42	46.43	1.80
340.00	1.11	1.16	8.80	47.37	2.04
360.00	1.08	1.16	8.24	46.15	2.19
380.00	1.08	1.12	8.18	47.06	2.40
400.00	1.07	1.15	8.18	47.67	2.62
420.00	1.07	1.16	8.09	47.27	2.84
440.00	1.09	1.16	8.07	50.00	3.13

Table S4. Branching ratios, in percentage, of the gas phase hydrogen abstraction from formic acid by ·OH in the 298.15-440 K temperature range.

**T (K)**	Γ_{Ia}	Γ_{Ib}	Γ_{IIa}	Γ_{IIb}
296.0	97.3	2.3	0.4	0.1
297.0	97.3	2.3	0.4	0.1
**298.15**	**97.1**	**2.4**	**0.4**	**0.1**
300.00	97.1	2.5	0.4	0.1
320.00	85.2	14.0	0.7	0.1
340.00	84.3	14.5	1.1	0.2
360.00	82.5	15.6	1.7	0.2
380.00	81.1	16.2	2.3	0.4
400.00	78.3	17.9	3.2	0.5
420.00	76.4	18.8	4.2	0.6
440.00	75.1	19.1	5.0	0.8

References

(1) Herzberg, G. *Molecular Spectra and Molecular Structure: Iii. Electronic Spectra and Electronic Structure of Polyatomic Molecules*, Von Nostrand Reinhold Co.: The USA, 1966; 745.

(2) Huber, K. P.; Herzberg, G. *Molecular Spectra and Molecular Structure: Iv. Constants of Diatomic Molecules*, Springer: The USA, 1979; 4, 716.

(3) Kim, E.; Bradforth, S.; Arnold, D.; Metz, R.; Neumark, D. Study of Hco2 and Dco2 by Negative Ion Photoelectron Spectroscopy. *J. Chem. Phys.* **1995**, *103*, 7801-7814.

(4) Dzib, E.; Cabellos, J. L.; Ortíz-Chi, F.; Pan, S.; Galano, A.; Merino, G. Eyringpy: A Program for Computing Rate Constants in the Gas Phase and in Solution. *Int. J. Quantum Chem.* **2019**, *119*, e25686.

(5) Duncan, W. T.; Bell, R. L.; Truong, T. N. Therate: Program for Ab Initio Direct Dynamics Calculations of Thermal and Vibrational-State-Selected Rate Constants. *J. Comput. Chem.* **1998**, *19*, 1039-1052.

(6) Canneaux, S.; Bohr, F.; Henon, E. Kisthelp: A Program to Predict Thermodynamic Properties and Rate Constants from Quantum Chemistry Results. *J. Comput. Chem.* **2014**, *35*, 82-93.

(7) Isaacson, A. D.; Truhlar, D. G. Polyatomic Canonical Variational Theory for Chemical Reaction Rates. Separable-Mode Formalism with Application to Oh+ H2→ H2o+ H. *J. Chem. Phys.* **1982**, *76*, 1380-1391.

(8) Isaacson, A. D.; Truhlar, D. G.; Rai, S. N.; Steckler, R.; Hancock, G. C.; Garrett, B. C.; Redmon, M. J. Polyrate: A General Computer Program for Variational Transition State Theory and Semiclassical Tunneling Calculations of Chemical Reaction Rates. *Comput. Phys. Commun.* **1987**, *47*, 91-102.

(9) Lu, D.-h.; Truong, T. N.; Melissas, V. S.; Lynch, G. C.; Liu, Y.-P.; Garrett, B. C.; Steckler, R.; Isaacson, A. D.; Rai, S. N.; Hancock, G. C.; Lauderdale, J. G.; Joseph, T.; Truhlar, D. G. Polyrate 4: A New Version of a Computer Program for the Calculation of Chemical Reaction Rates for Polyatomics. *Comput. Phys. Commun.* **1992**, *71*, 235-262.

(10) Ferro-Costas, D.; Truhlar, D. G.; Fernández-Ramos, A. Pilgrim: A Thermal Rate Constant Calculator and a Chemical Kinetics Simulator. *Comput. Phys. Commun.* **2020**, *256*, 107457.

(11) Zhang, R. M.; Xu, X.; Truhlar, D. G. Tumme: Tsinghua University Minnesota Master Equation Program. *Comput. Phys. Commun.* **2022**, *270*, 108140.
